# Increased fluctuation in a butterfly metapopulation leads to diploid males and decline of a hyperparasitoid

**DOI:** 10.1098/rspb.2018.0372

**Published:** 2018-08-22

**Authors:** Abhilash Nair, Etsuko Nonaka, Saskya van Nouhuys

**Affiliations:** 1 Metapopulation Research Centre, Department of Biosciences, University of Helsinki, PO Box 65, 00014 Helsinki, Finland; 2 Department of Ecology, Environment and Plant Sciences, Stockholm University, Stockholm, 114 18, Sweden; 3 Department of Entomology, Cornell University, Ithaca, NY 14853, USA

**Keywords:** bottleneck, climate change, complementary sex determination, host–parasitoid dynamics, diploid male vortex, extinction

## Abstract

Climate change can increase spatial synchrony of population dynamics, leading to large-scale fluctuation that destabilizes communities. High trophic level species such as parasitoids are disproportionally affected because they depend on unstable resources. Most parasitoid wasps have complementary sex determination, producing sterile males when inbred, which can theoretically lead to population extinction via the diploid male vortex (DMV). We examined this process empirically using a hyperparasitoid population inhabiting a spatially structured host population in a large fragmented landscape. Over four years of high host butterfly metapopulation fluctuation, diploid male production by the wasp increased, and effective population size declined precipitously. Our multitrophic spatially structured model shows that host population fluctuation can cause local extinctions of the hyperparasitoid because of the DMV. However, regionally it persists because spatial structure allows for efficient local genetic rescue via balancing selection for rare alleles carried by immigrants. This is, to our knowledge, the first empirically based study of the possibility of the DMV in a natural host–parasitoid system.

## 1. Introduction

The frequency of weather extremes is increasing under ongoing climate change [1]. One repercussion of extreme events is increased spatial synchrony of local populations [2], which can decrease stability regionally and alter biotic interactions and community structure [3]. Small populations are vulnerable to a cascading feedback between demography and loss of genetic diversity, leading to extinction via the extinction vortex [4]. The risk of extinction increases especially in species at high trophic levels, as resources they depend on become increasingly sparse and locally unstable [5,6]. As resources become scarce, declining populations may lose genetic diversity, further increasing the risk of extinction [7,8]. In insect communities, parasitoids occupy the higher trophic levels. They interact strongly with their hosts, and those with limited host ranges have smaller population sizes than do their hosts [9].

**Figure 1.**
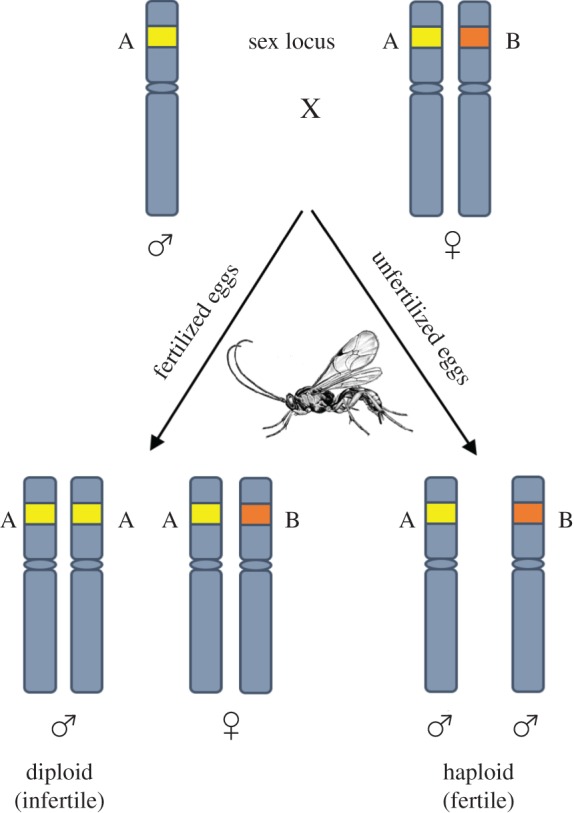
A schematic representation of single locus complimentary sex determination.

Parasitoid wasps lay eggs in or on other arthropods. The larvae then consume and eventually kill their hosts. As all other Hymenoptera, they are haplodiploid. Females are diploid, developing from fertilized eggs, and males are haploid, developing from unfertilized eggs. Inbreeding can be detrimental for haplodiploid species that have single locus complementary sex determination (sl-CSD). In these species sex is determined by a single locus, wherein haploid individuals develop as males and diploid individuals develop as females when heterozygous [10]. Under sl-CSD, inbreeding or matched mating results in half the fertilized eggs being homozygous at the sex locus and developing into diploid males at the expense of female offspring (figure 1).

Diploid males are inviable [11] with a few exceptions [12,13]. Thus, production of diploid males is costly to the parents, and in the case of sterile diploid males, to their mates [14,15]. sl-CSD is the most prevalent and presumably the ancestral mode of sex determination in Hymenoptera [10,16,17]. Many parasitoid species produce diploid males under laboratory conditions (e.g. [12,18–21]). They are also found in natural populations [20,22] and after introduction for biological control [23–25]. Zayed & Packer [26] demonstrated theoretically that high production of diploid males in small and isolated populations of species with sl-CSD can elevate extinction risk through a ‘diploid male vortex’ (DMV), in which accelerating decline in population size triggers positive feedback between small population size and loss of alleles at the CSD locus. The DMV increases the risk of extinction in haplodiploid over diploid species living in similar ecological circumstances. Later theoretical studies challenged the assumptions made by Zayed & Packer [26], showing that DMVs require a set of stringent conditions if relevant behavioural, demographic, and ecological factors are included [27–29]. Particularly, a small amount of dispersal in spatially structured populations promotes the maintenance of CSD alleles through strong balancing selection for rare alleles [27,30]. In single-species population dynamic models incorporating CSD [19,25–27,29], resource availability (carrying capacity) is kept constant over time, but large population fluctuations could cause genetic bottlenecks leading to loss of CSD alleles from the population [25]. The existing models have not incorporated both temporal host population fluctuations (fluctuation in resource availability) and spatial population structure. Such extensions are necessary to understand how populations constrained by CSD may fair with increased habitat fragmentation and fluctuations in environmental conditions that are anticipated under climate change.

Existing empirically based studies of diploid male production (DMP) do not come to a consensus on its significance for the persistence of natural populations [24,31–34]. So far, evidence for DMVs occurring in the wild has not been demonstrated. To do so, sampling would have to be done over time. We studied DMP in a hyperparasitoid that is part of the insect community associated with the Glanville fritillary butterfly, *Melitaea cinxia* (Lepidoptera: Nymphalidae) in the Åland islands, southwest Finland [35], over a period of four years. The host butterfly lives as a classic metapopulation inhabiting 300–500 small meadows over an area of 3500 km^2^ [36,37]. In recent years, spatial synchrony of the local butterfly population sizes has increased in Åland. This appears to be owing to increasing extreme and spatially synchronized weather, leading to spatial autocorrelation of population sizes. This synchrony is due to local rates of survival and reproduction and not because of homogenizing dispersal in the landscape [38]. The increasing butterfly population fluctuation must affect the community of parasitoids that depend on it, and indeed, we have observed a decline in the effective population size (*N*_e_) of the hyperparasitoid [39]. Here, we address the population level consequences of DMP for the hyperparasitoid, using empirical data from large-scale field sampling over time and a spatially-structured multitrophic population model. Our study demonstrates the long-term impact of increased fluctuations in the population dynamics of the host butterfly (the second trophic level) on the population size of a hyperparasitoid (the fourth trophic level), mediated through both demographic processes and loss of genetic diversity at the CSD locus.

## 2. Material and methods

### (a) Study species

**Figure 2.**
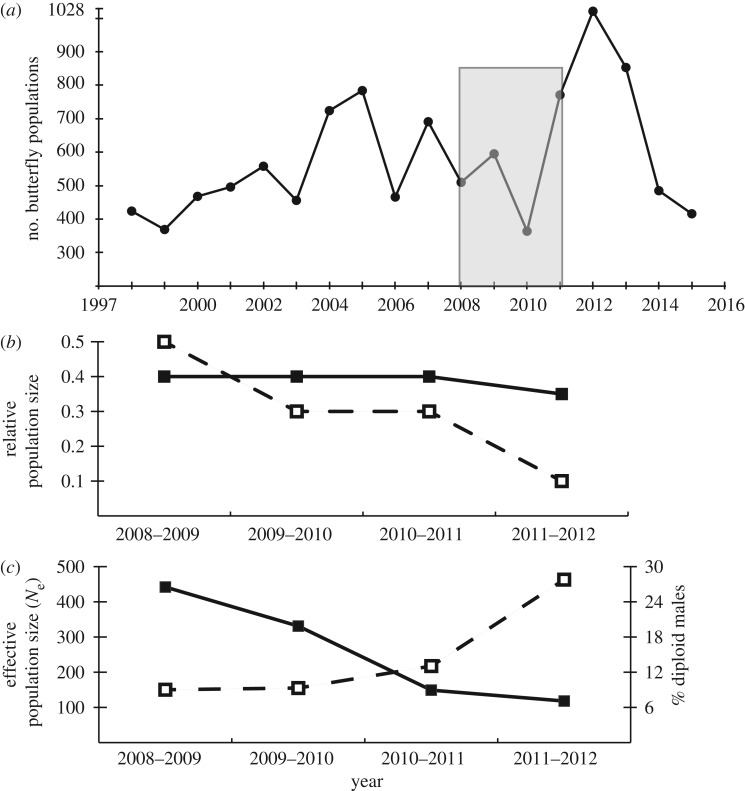
(*a*) The metapopulation size of the butterfly measured as the number of local populations from 1997 to 2015. The shaded box indicates the focal years for this study. (*b*) The population size of the primary parasitoid relative to the butterfly (solid line), and the population size of the hyperparasitoid (dashed line) relative to the host parasitoid from 2008–2009 to 2011–2012. The parasitoid population sizes are estimated from the rate of parasitism by each species in the butterfly samples 2008–2011. The butterfly population size is from the annual survey [36]. (*c*) The effective population size (*N*_e_) of the hyperparasitoid (left axis, solid line) [39] and the percentage of diploid males that were in samples from year 2008–2009 to 2011–2012 (right axis, dashed line).

*Mesochorus* cf. *stigmaticus* (Hymenoptera: Ichneumonidae) is a hyperparasitoid wasp that lays eggs only into larval parasitoids developing inside *M. cinxia* caterpillars. The wasp is found throughout the butterfly metapopulation in Åland [39], where it uses almost exclusively the host *Hyposoter horticola* (Hymenoptera: Ichneumonidae) which is a specialized solitary egg-larval parasitoid of the butterfly [35,40]. A peculiarity of this system is that the primary parasitoid (*H. horticola*), under natural conditions consistently parasitizes about a third of the butterfly larvae in each gregarious nest (figure 2*b*). Along with its strong dispersal ability [41], this behaviour translates to a uniform rate of parasitism over the landscape independent of local or regional host density [40,42]. As a result of the lack of local density dependence of the *primary* parasitoid, the population dynamics of the butterfly can be considered as directly influencing the *hyper*parasitoid.

### (b) Sample collection, rearing and genotyping

*Melitaea cinxia* larvae live gregariously in silk nests. Three larvae, some of which were naturally parasitized and hyperparasitized, were collected from each nest during the annual autumn survey of Åland [36] over four years (autumn 2008–2011). The number of nests sampled ranged from 1224 to 4689 owing to the yearly fluctuation of butterfly population size. The collected larvae were reared in the laboratory until they became butterflies, parasitoids emerged from them, or they died. The number of adult hyperparasitoids reared differed between years (*n* = 48–361), depending on the host sample size, overwintering mortality, and the rate of hyperparasitism. Adult wasps were stored in 96% ethanol at −20°C for genetic analysis. The samples were used to estimate the rates of parasitism and hyperparasitism in the population, and to determine the fraction of hyperparasitoid males that were diploid. DNA was extracted from tissue of males using DNeasy isolation kit (Qiagen, Hilden, Germany), and genotyped using species-specific microsatellite loci [43]. The detailed genotyping methods are described in Nair *et al*. [39].

#### (i) Data analyses

Diploid males were identified based on genotyping results from 17–25 microsatellite markers [43]. An individual was identified as a diploid male if it lacked an ovipositor, and one or more microsatellite marker was heterozygous. The percentage of diploid male for each year was estimated as the fraction of males that were diploid in the field samples. The number of hyperparasitoid migrants per generation between survey areas was estimated in Genepop (ver. 4.2) [44] using neutral microsatellite data (total wasps = 175) from Nair *et al*. [45].

### (c) The model

We developed a discrete-time individual-based simulation model of spatially structured populations of a hyperparasitoid including explicit genetics for CSD. It simulates the population dynamics of the hyperparasitoid in response to fluctuation and spatial autocorrelation of local butterfly populations. The model is formulated using the known long-term butterfly dynamics [36,38] and parasitoid population dynamics, genetic structure and natural history [35,39,40]. Table 1 lists the state variables (*a*) and model parameters (*b*). More detail can be found in the electronic supplementary material. The model was implemented in Matlab R2017a. The code is available from the Dryad Digital Repository: https://doi.org/10.5061/dryad.56qf11h [46].

**Table 1.** The state variables (*a*) and parameters (*b*) in the model. (Subscripts *i* and *t* signify sub-regions and time step (generation), respectively. See the electronic supplementary material, table S1 for the full description of the model and details of the state variables and parameters.)

	description
(*a*) state variables	
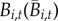	butterfly larval population size (mean)
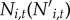	parasitoid population size after (before) hyperparasitism
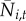	mean parasitoid population size before hyperparasitism
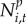	number of hyperparasitized parasitoids
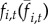	mean rate of hyperparasitism
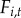	number of female hyperparasitoids
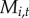	number of male hyperparasitoids
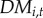	number of diploid male hyperparasitoids
(*b*) model parameters	
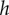	migration rate = 0.002, 0.014, 0.027
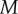	multiplier on the standard deviation of larval population size
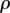	mean cross-correlation of larval population size between survey areas
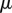	mutation rate at the CSD locus = 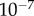

#### (i) The spatial structure in the model

The study area was divided into 12 sub-regions, based on the butterfly survey areas (electronic supplementary material, figure S1; [36]) to represent realistic heterogeneity in host availability to the hyperparasitoid arising from spatial distribution of habitat patches for the butterfly. The hyperparasitoid is dispersive [39] but individual wasps (approximately 6 mm in size) are dispersal limited and their distribution in the landscape is limited by the heterogeneity of host availability [35]. We estimated the butterfly larval abundances annually using the autumn survey [36] data from 2003 to 2016. The populations in four sub-regions were designated as small (average number of larvae < 3000), four were medium (between 3000 and 6000), and four were large (greater than 10 000). To account for uncertainty in dispersal patterns of the hyperparasitoid across sub-regions, the sub-regions were randomly arranged in a 3 × 4 lattice in each simulation with links in cardinal directions (the sub-regions on edges had fewer links).

#### (ii) The butterfly larvae sub-model

The mean population size of butterfly larvae at time *t* in sub-region *i*, 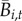, is drawn from a multivariate log-normal distribution with the means and the variance–covariance matrix of the associated multivariate normal distribution estimated from the annual autumn survey data. The time step is 1 year (generation) and, as until recently in the survey data, there is no temporal correlation between years [38]. The actual number of larvae in sub-region *i*, 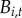, was determined by drawing values from a Poisson distribution with mean equal to 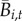. Based on the survey data, the estimates for the correlation of nest counts among the sub-regions ranged from about 0.25 to 0.75, and the standard deviations increased 1.85-fold between 2003–2009 and 2010–2016. The spatial variation was due primarily to spatially uncorrelated weather [38]. We simulated variability in butterfly population fluctuations by varying the cross-correlation coefficient, 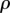, and the factor *M* multiplying the standard deviations in calculating the variance–covariance matrix for population dynamics in the sub-regions.

#### (iii) The host parasitoid sub-model

The population size of the parasitoid before hyperparasitism at time *t* in sub-region *i*, 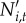, is drawn from a binomial distribution with the butterfly larval population size as the sample size and probability of success equal to 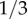 [40,42]. We modelled the mean rate of hyperparasitism at time *t* in sub-region *i*, 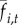, as a saturating function of the ratio between hyperparasitoid female and parasitoid densities, and visually fit the function to empirical data (electronic supplementary material, E1 and figure S2). We used the data as a guide to estimate the parameters because the functional form is theoretically reasonable and available data are limited. To reflect yearly stochastic variation, the actual rate of hyperparasitism was drawn from a normal distribution with the mean equal to 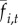 with standard deviation 0.1 to match the variation seen in the empirical data (electronic supplementary material, E2 and figure S2). The number of parasitized hosts at time *t*, 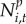 (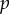 for parasitized) is drawn from a binomial distribution with probability of success equal to 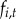 and the number of trials equal to the number of parasitoids.

#### (iv) The hyperparasitoid sub-model

In the model, each individual hyperparasitoid is represented by one sex locus. We assume diploid males to be sterile. An individual may disperse once with equal probability *h* (migration rate) to any directly neighbouring sub-region (two-dimensional stepping-stone dispersal [47,48]). We used three levels of *h*, 0.002, 0.014, and 0.027 (electronic supplementary material, table S1) to account for the uncertainty and variation of migration rate observed in the temporal genetic structure of the wasp [39]. After the dispersal step, the hyperparasitoid mates in the natal or destination sub-region. Mates are randomly paired and polyandry is assumed. The total number of offspring at time *t* in sub-region *i* is 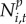. The parental genotypes (sl-CSD allele(s)) are randomly assigned to each offspring and passed on according to Mendelian inheritance for haplodiploidy. Each allele mutates into a new allele with a probability 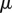 of 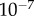 [25,49].

#### (v) Simulation experiments

We ran 30 replicates for each parameter set for 10 000 generations to ensure that transient dynamics disappeared and that sufficient numbers of local extinctions were observed to distinguish decline owing to demographics from that owing to CSD. We initialized the population with 10 alleles at the CSD locus, randomly distributed among hyperparasitoid individuals. We chose 10 to achieve [17,39,50] the 9–10% of diploid males present at the low level of fluctuation in butterfly population size observed between 2003 and 2009. We varied the multiplier on the level of fluctuation, *M*, to cover a parameter region from zero to three times the baseline (2009) fluctuation of the butterfly population sizes. The correlation parameter 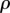 was varied from 0 to 1. We summarized outputs from the last 5000 generations at the whole Åland scale and at the sub-regional scale. To isolate the consequences of DMP from demographic effects of host population dynamics, we compared the model outcomes under sl-CSD conditions with those from a hypothetical scenario in which all diploid offspring develop normally as female. We expressed the costs due to DMP by taking the difference between the two scenarios with respect to extinction rate, total population size, and the number of persisting sub-regions.

## 3. Results

### (a) Population dynamics and population genetics of the natural populations

The metapopulation size of the host butterfly, and population sizes of the primary parasitoid, and the hyperparasitoid were the lowest ever recorded in 2010–2011 (figure 2*a*). The population size of the hyperparasitoid relative to that of the butterfly declined over the four years of the study, whereas that of the primary parasitoid remained constant. In 2011–2012, the relative population size of the hyperparasitoid decreased sharply despite rapid recovery of the butterfly (figure 2*b*). Using microsatellite markers, we found that among the collected hyperparasitoids the percentage of males that were diploid increased from about 9% in 2008–2009 and 2009–2010, to 13% in 2010–2011, and 28% in 2011–2012. The hyperparasitoid *N*_e_ also declined steeply [39] (figure 2*c*).

### (b) Simulation experiments

**Figure 3.**
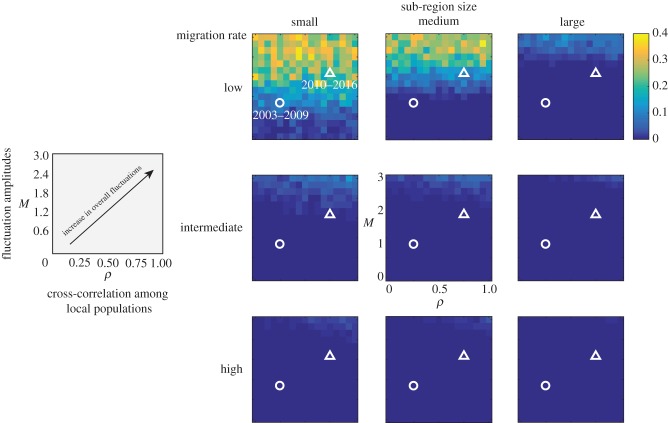
The cost of diploid male production by the hyperparasitoid in small, medium, and large sub-regions, independent of demographic effects, with varying cross-correlation among local populations 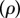 and fluctuation multiplier (*M*), at three levels of migration rate. The cost (an increase in extinction rate) was quantified as the difference in extinction rate between the scenarios with CSD on and off. The extinction rate is defined as the probability of extinction given that the population was present in the previous year. Extinction is very rare without CSD. The levels of fluctuation in the data for two periods are shown as a circle for 2003–2009 and a triangle for 2010–2016.

The results of the simulation model illustrate that DMP is costly for the hyperparasitoid, over and above the direct demographic costs of fluctuating butterfly abundance at high fluctuation amplitudes and at low and intermediate migration rate (figure 3). The effect of the cross-correlation among local populations is smaller than that of fluctuation amplitude (M). Small sub-regions become locally extinct (figure 3), lose sex alleles (electronic supplementary material, figure S3A), and have increased DMP at lower levels of host population fluctuation (electronic supplementary material, figure S3B).

**Figure 4.**
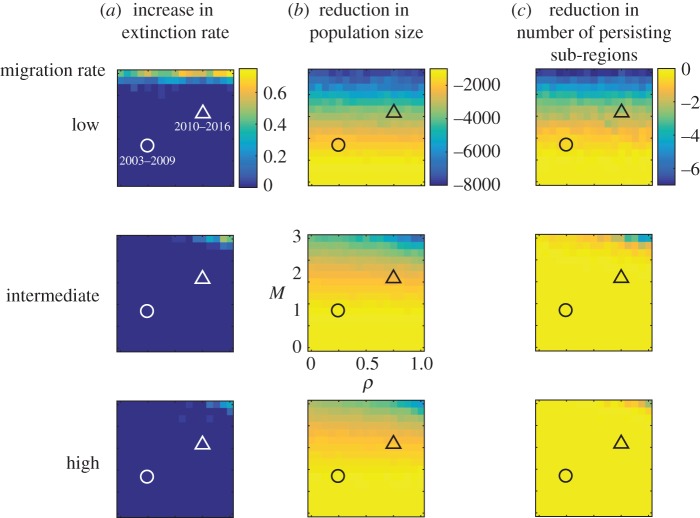
The cost of diploid male production by the hyperparasitoid at the whole Åland scale*,* independent of demographic effects, with varying cross-correlation among local populations 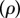 and fluctuation multiplier (*M*), at three levels of migration rate. The cost was quantified as the difference between the scenarios with CSD on and off as an increase in extinction rate (*a*), a reduction in population size (the total number of individuals) (*b*), or a reduction in the number of persisting local populations (*c*). The extinction rate is the proportion of simulation runs that ended with global extinction, out of 30 replicates. The levels of fluctuations in the data for two periods are shown as a circle for 2003–2009 and as a triangle for 2010–2016. Total population size and the number of persisting sub-regions are negative because these were lower with CSD turned on.

**Figure 5.**
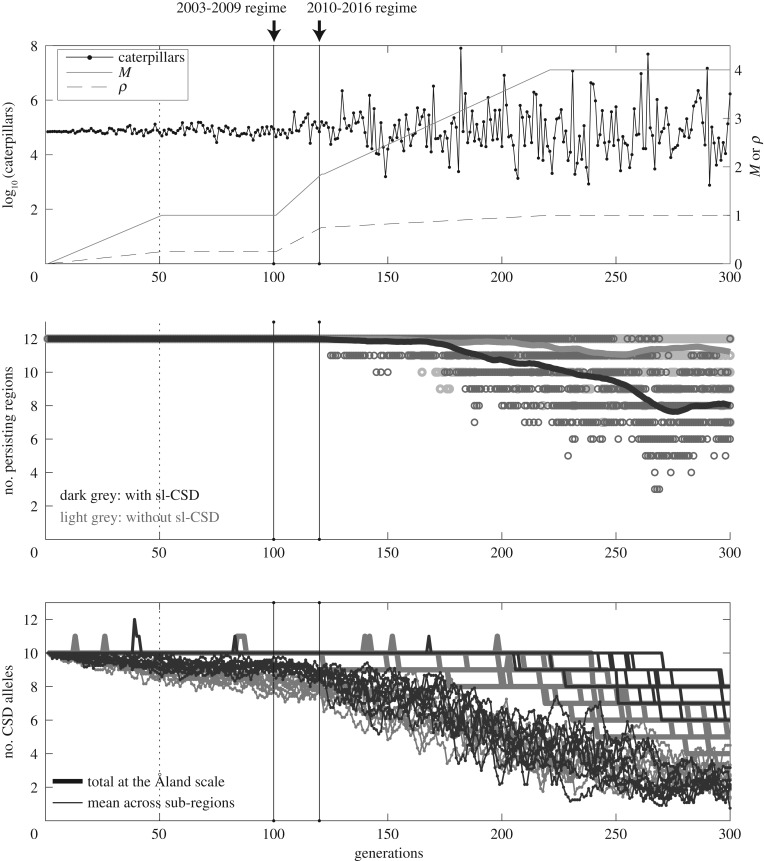
An illustration of a hypothetical scenario in which fluctuation and synchrony of the butterfly population continuously increase over time. Migration rate of the hyperparasitoid was intermediate (*h* = 0.014). The conditions at the two black vertical lines correspond to the empirical 2003–2009 and 2010–2016 butterfly population dynamics. (*a*) The butterfly population size (left axis) and temporal changes in the two parameters controlling population fluctuation, *M* and *p* (right axis). (*b*) The number of persisting sub-regions when sl-CSD is present (dark grey) and absent (light grey) from 10 replicate runs. Each circle represents the number of persisting sub-regions. The thick lines are moving window averages of circles with a corresponding colour. (*c*) Thin lines: the mean number of sl-CSD alleles across sub-regions with (dark grey) and without (light grey) CSD. Thick lines: the number of unique sl-CSD alleles in the whole Åland.

Though locally sex alleles are lost, overall allelic diversity is maintained in the whole Åland even with relatively high host population fluctuation, and extinction of the hyperparasitoid is unlikely (electronic supplementary material, figure S4A-b). Once the amplitude of butterfly population fluctuation and autocorrelation surpass what has been observed in the natural system, the costs of DMP increase greatly (figures 4 and 5). Fewer sex alleles are maintained, which increases diploid male production. Overall population size declines, resulting in fewer local sub-regions persisting (electronic supplementary material, figure S4A). This pattern is most prominent at low hyperparasitoid migration rate.

The importance of balancing selection in maintaining sex alleles in the landscape is illustrated by comparing the number of alleles under sl-CSD (dark grey lines, figure 5*c*) with the allele number when all diploids are females (light grey lines, figure 5*c*). In the latter case, genetic drift owing to demographic stochasticity causes a large loss of allelic diversity without balancing selection, as host population size increasingly fluctuates.

## 4. Discussion

We present a scenario in which large fluctuations in population dynamics of a herbivore due to environmental change propagate up through the food chain, affecting a 4th-trophic-level hyperparasitoid, both demographically and genetically. In addition, we show the importance of spatial population structure and genetic rescue among local populations for persistence under rapid environmental change. During a period of increased fluctuation of the host metapopulation size, we detected a precipitous increase in production of diploid males by the hyperparasitoid, up to approximately 28%, with a decline in population size that is evocative of a DMV. Our simulation model suggests that, despite signs of a DMV, the hyperparasitoid may not be facing regional extinction because of genetic rescue, which depends on spatial population structure and strong balancing selection resulting in global maintenance of high allelic diversity at the hyperparasitoid sex locus.

### (a) The burden of single locus complementary sex determination and countermeasures

sl-CSD can be a constraint for haplodiploid species because inbreeding causes the production of inviable or sterile diploid males [10]. Many Hymenoptera minimize the cost of sl-CSD by avoiding inbreeding behaviourally via natal dispersal, protandry, or mate choice [51–53]. Other parasitoid species have evolved multilocus (ml)-CSD to reduce DMP [18,23] or evolved means to produce fertile diploid males [12,13]. Some parasitoids have developed completely different sex determination mechanisms (e.g. [54–56]). The relatively frequent evolution of countermeasures to DMP suggests strong selection against it in the evolutionary history of these species. The hyperparasitoid in this system is dispersive, as shown by its wide distribution in the landscape and very low spatial genetic structure relative to the host parasitoid and the host butterfly [39]. Being dispersive should reduce inbreeding and reduce diploid male load [27,29]. Thus, it may be that under normal conditions this wasp minimizes the costs of sl-CSD through dispersal.

We observed a relatively high percentage of diploid males (about 9%) despite a large *N*_e_ before the wasp declined (figures 2 and 3). This suggests that the wasp has sl-CSD, rather than ml-CSD [25]. On islands species often have reduced genetic diversity owing to isolation and founder effects [57–59]. Thus, we expect Hymenoptera with CSD on islands to have relatively high DMP [20]. For example, captive (9.5%) and natural island populations (12%) of the parasitoid *Venturia canescens* have higher DMP than mainland populations (4.3%) [20].

In order to detect the cost of DMP and find evidence of DMVs, data should be collected over time to show an increase in DMP along with decreasing population size or *N*_e_. In one of the few studies encompassing more than a single season Weis *et al.* [25] followed up on a study by de Boer *et al.* [23] of an introduced population of the parasitoid *Cotesia rubecula.* They show that, despite a population bottleneck upon introduction, DMP decreased from 8–13% to 0–3% over 10 years. Additionally, balancing selection maintained high variation at the CSD locus. Similarly, Gloag *et al.* [30] saw the restoration of allelic diversity at the CSD loci during the invasion into Australia of the Asian honeybee. Unlike these studies, we found an increase in the percentage of diploid males in the hyperparasitoid with a drastic decline in *N*_e_ over the period of four years, indicating the possibility of an ongoing DMV.

### (b) Host population fluctuations and diploid male vortices in the hyperparasitoid

The metapopulation size of the host butterfly in Åland has historically been relatively constant, although local populations fluctuate strongly [36]. The hyperparasitoid has tolerated these local fluctuations well via dispersive foraging that allows it to find hosts even in newly colonized local butterfly populations [35,39]. Over the last decade, the butterfly metapopulation has experienced increased spatial synchrony of local population sizes and fluctuation of population size that is attributable to increased weather extremes [38]. As part of this trend, 2010–2011 had the lowest recorded metapopulation size over the last 20 years (figure 2*a*).

The primary parasitoid maintained a constant rate of parasitism (about 1/3 of the butterfly population) over the four years of the study (figure 2*b*). By contrast, the relative population size of the hyperparasitoid declined (figure 2*c*). In addition, its absolute neutral allelic diversity declined, while spatial genetic structure increased with decreasing population size [39]. In fact, the hyperparasitoid population did not recover in spite of the dramatic recovery of the butterfly and primary parasitoid populations in 2011–2012 (figure 2*b*). These observations suggest that some sex alleles were lost or became very rare during the 2010–2011 crash. The sharp decline in the hyperparasitoid along with exceptionally high frequencies of diploid males is in accordance with the theoretical predictions of the DMV [26].

Small populations are at severe risk of extinction simply because of demographic and environmental stochasticity [60] as well as genetic factors [8,61]. Our model results elucidate the cost of DMP in the hyperparasitoid separate from demographic extinction risks. To illustrate the potential effects of climate changes, we gradually increased spatial synchrony and variability of fluctuations in local butterfly population size in the model from completely asynchronous with minimal fluctuation to highly synchronous with large fluctuation (figure 5*a*). Under high fluctuation of the butterfly population, the hyperparasitoid experiences increased local extinctions associated with increased DMP, hence a DMV, in small- to medium-sized sub-regions of the landscape (figures 3 and 5). Regional extinction risk owing to a DMV increase only at fluctuation rates well beyond what has been observed in Åland (figure 4). At the current level of fluctuation (years 2010–2017), the hyperparasitoid population is unlikely to be facing an imminent extinction from the entire Åland islands. In fact, the hyperparasitoid still persists now in 2018. On the other hand, at the sub-region scale the number of sex alleles declines, the fraction of diploid males increases, and total population sizes decrease even under the current conditions, which could explain why the hyperparasitoid population continued to decline despite the recovery of the butterfly.

### (c) Genetic rescue and balancing selection

Balancing selection can reduce the proportion of diploid males in a population over time by maintaining high allelic diversity at CSD loci [25,30]. Dispersal of individuals between sub-regions can rescue local populations from extinction both demographically and genetically [62,63]. Dispersal together with balancing selection in a spatially structured population may be sufficient to prevent a DMV [27]. Genetic rescue is particularly effective when unique sex alleles are maintained in different parts of the landscape. In our model local hyperparasitoid populations collectively maintain all 10 sex alleles, and local populations in larger sub-regions are able to maintain a large fraction of them when conditions are not extreme (figure 5; electronic supplementary material, figure S4). Hence, our study generalizes the earlier finding by Hein *et al.* [27] to highly fluctuating host and parasitoid populations. The effectiveness of this process is illustrated by the sharp contrast between the maintenance of sex alleles and rapid loss and fixation of a neutral allele through genetic drift under the same conditions (figure 5*c*; electronic supplementary material, figure S4A-b, S4B-b). The reduction of population decline caused by inbreeding via maintenance of allelic diversity through balancing selection is not confined to Hymenoptera with CSD. For instance, it is also found in plant species that have the gametophytic self-incompatibility S-locus [64,65].

Zayed & Packer [26] showed that theoretically sl-CSD increases extinction rate of haplodiploid populations leading to a DMV. Later theoretical studies relaxed the assumptions that populations are small and isolated, but most of the models assume resource (host) availability to the parasitoid to be temporally constant [25–27,29]. Bompard *et al.* [28] made a non-spatial host–parasitoid model that allowed for temporal fluctuation of host density arising from the explicit host–parasitoid interaction. They found DMP to stabilize rather than destabilize the parasitoid population by dampening endogenous cyclic fluctuations, which made DMVs less likely. Our model includes both temporal and spatial fluctuation of host availability caused by exogenous (i.e. environmental) fluctuations and spatial heterogeneity. In addition, we show that the occurrence of DMV-driven extinctions depends on the spatial scale: DMP elevates extinction risk in small local populations via a DMV, but the risk is substantially alleviated in a spatially structured population via genetic rescue and strong balancing selection. As many populations are spatially structured in nature [66], DMVs appear not to be as likely in natural populations as it was proposed by Zayed & Packer [26]. However, once a population enters a DMV initiated by climate change, habitat fragmentation, or pesticide use (in the case of bees), the trajectory towards extinction might proceed quickly. Monitoring for harbingers of a DMV, such as increasing proportions of diploid males [67], may be a good monitoring strategy, as larger environmental fluctuations are anticipated under climate change and there are many ecologically and economically important species of Hymenoptera.

## Ethics

There are no ethical issues associated with species used in this research.

## Data accessibility

The data supporting the results and Matlab code are archived in the Dryad Digital Repository at: http://dx.doi.org/10.5061/dryad.56qf11h [46].

## Authors' Contributions

S.v.N. and A.N. conceived and designed the study. A.N. conducted the molecular laboratory work. E.N. designed and developed the model and conducted the simulation experiments. A.N., E.N. and S.v.N. did the data analysis and wrote the manuscript. All authors gave final approval for publication.

## Competing interests

We have no competing interests.

## Funding

The project was funded by the Academy of Finland (no. 125553 to S.v.N., and nos. 129636 and 134746 to I. Hanski), the Finnish Cultural Foundation (A.N.), and the Swedish Research Council (E.N.).

## Supplementary Material

Model description

## References

[1] Coumou D, Rahmstorf S 2012 A decade of weather extremes. Nat. Clim. Change 2, 491–496. (10.1038/nclimate1452)

[2] Liebhold A, Koenig WD, Bjornstad ON 2004 Spatial synchrony in population dynamics. Annu. Rev. Ecol. Evol. Syst. 35, 467–490. (10.1146/annurev.ecolsys.34.011802.132516)

[3] Hansen BB, Grøtan V, Aanes R, Sæther B-E, Stien A, Fuglei E, Ims RA, Yoccoz NG, Pedersen ÅØ 2013 Climate events synchronize the dynamics of a resident vertebrate community in the high Arctic. Science 339, 313–315. (10.1126/science.1226766) 23329044

[4] Gilpin ME, Soulé ME 1986 Minimum viable populations: processes of species extinction. In Conservation biology: the science of scarcity and diversity (ed Soulé ME), pp. 19–34. Sunderland, MA: Sinauer Associates.

[5] Holt RD 2002 Food webs in space: on the interplay of dynamic instability and spatial processes. Ecol. Res. 17, 261–273. (10.1046/j.1440-1703.2002.00485.x)

[6] Voigt W et al. 2003 Trophic levels are differentially sensitive to climate. Ecology 84, 2444–2453. (10.1890/02-0266)

[7] Keller LF, Waller DM 2002 Inbreeding effects in wild populations. Trends Ecol. Evol. 17, 230–241. (10.1016/S0169-5347(02)02489-8)

[8] Spielman D, Brook BW, Frankham R 2004 Most species are not driven to extinction before genetic factors impact them. Proc. Natl Acad. Sci. USA 101, 15 261–15 264. (10.1073/pnas.0403809101) PMC524053 15477597

[9] Hassell MP 2000 The spatial and temporal dynamics of host-parasitoid interactions. London, UK: Oxford University Press.

[10] Heimpel GE, de Boer JG 2008 Sex determination in the Hymenoptera. Annu. Rev. Entomol. 53, 209–230. (10.1146/annurev.ento.53.103106.093441) 17803453

[11] Zayed A 2004 Effective population size in Hymenoptera with complementary sex determination. Heredity 93, 627–630. (10.1038/sj.hdy.6800588) 15354193

[12] Cowan DP, Stahlhut JK 2004 Functionally reproductive diploid and haploid males in an inbreeding hymenopteran with complementary sex determination. Proc. Natl Acad. Sci. USA 101, 10 374–10 379. (10.1073/pnas.0402481101) PMC478579 15232002

[13] Zaviezo T, Retamal R, Urvois T, Fauvergue X, Blin A, Malausa T 2018 Effects of inbreeding on a gregarious parasitoid wasp with complementary sex determination. Evol. Appl. 11, 243–253. (10.1111/eva.12537) 29387159 PMC5775491

[14] MacBride DH 1946 Failure of sperm of *Habrobracon* diploid males to penetrate the eggs. Genetics 31, 224. 21021055

[15] de Boer JG, Ode PJ, Vet LEM, Whitfield J, Heimpel GE 2007 Diploid males sire triploid daughters and sons in the parasitoid wasp *Cotesia vestalis*. Heredity 99, 288–294. (10.1038/sj.hdy.6800995) 17551527

[16] Cook JM, Crozier RH 1995 Sex determination and population biology in the Hymenoptera. Trends Ecol. Evol. 10, 281–286. (10.1016/0169-5347(95)90011-X) 21237037

[17] Harpur BA, Sobhani M, Zayed A 2012 A review of the consequences of complementary sex determination and diploid male production on mating failures in the Hymenoptera. Entomol. Exp. Appl. 146, 156–164. (10.1111/j.1570-7458.2012.01306.x)

[18] de Boer JG, Ode PJ, Rendahl AK, Vet LEM, Whitfield JB, Heimpel GE 2008 Experimental support for multiple-locus complementary sex determination in the parasitoid *Cotesia vestalis*. Genetics 180, 1525–1535. (10.1534/genetics.107.083907) 18791258 PMC2581954

[19] Fauvergue X, Chuine A, Vayssade C, Auguste A, Desouhant E 2015 Sterile males in a parasitoid wasp with complementary sex determination: from fitness costs to population extinction. BMC Ecol. 15, 13 (10.1186/s12898-014-0032-6) 25962498 PMC4449571

[20] Collet M, Vayssade C, Auguste A, Mouton L, Desouhant E, Malausa T, Fauvergue X 2016 Diploid male production correlates with genetic diversity in the parasitoid wasp *Venturia canescens*: a genetic approach with new microsatellite markers. Ecol. Evol. 6, 6721–6734. (10.1002/ece3.2370) 27777743 PMC5058541

[21] Retamal R, Zaviezo T, Malausa T, Fauvergue X, Le Goff I, Toleubayev K 2016 Genetic analyses and occurrence of diploid males in field and laboratory populations of *Mastrus ridens* (Hymenoptera: Ichneumonidae), a parasitoid of the codling moth. Biol. Control 101, 69–77. (10.1016/j.biocontrol.2016.06.009)

[22] Ruf D, Dorn S, Mazzi D 2013 Unexpectedly low frequencies of diploid males in an inbreeding parasitoid with complementary sex determination. Biol. J. Linn. Soc. 108, 79–86. (10.1111/j.1095-8312.2012.01976.x)

[23] de Boer JG, Kuijper B, Heimpel GE, Beukeboom LW 2012 Sex determination meltdown upon biological control introduction of the parasitoid *Cotesia rubecula*? Evol. Appl. 5, 444–454. (10.1111/j.1752-4571.2012.00270.x) 22949920 PMC3407863

[24] de Boer JG, Groenen MA, Pannebakker BA, Beukeboom LW, Kraus RH 2015 Population-level consequences of complementary sex determination in a solitary parasitoid. BMC Evol. Biol. 15, 98 (10.1186/s12862-015-0340-2) 26025754 PMC4461988

[25] Weis JJ, Ode PJ, Heimpel GE 2017 Balancing selection maintains sex determining alleles in multiple-locus complementary sex determination. Evolution 71, 1246–1257. (10.1111/evo.13204) 28225571

[26] Zayed A, Packer L 2005 Complementary sex determination substantially increases extinction proneness of haplodiploid populations. Proc. Natl Acad. Sci. USA 102, 10 742–10 746. (10.1073/pnas.0502271102) PMC1180771 16020532

[27] Hein S, Poethke HJ, Dorn S 2009 What stops the ‘diploid male vortex’? A simulation study for species with single locus complementary sex determination. Ecol. Modell. 220, 1663–1669. (10.1016/j.ecolmodel.2009.04.001)

[28] Bompard A, Amat I, Fauvergue X, Spataro T 2016 Trophic interactions may reverse the demographic consequences of inbreeding. Ecology 97, 3131–3142. (10.1002/ecy.1544) 27870041

[29] Faria LRR, Soares EDG, do Carmo E, de Oliveira PMC 2016 Diploid male dynamics under different numbers of sexual alleles and male dispersal abilities. Theory Biosci. 135, 111–119. (10.1007/s12064-016-0226-x) 27067711

[30] Gloag R, Ding G, Christie JR, Buchmann G, Beekman M, Oldroyd BP 2016 An invasive social insect overcomes genetic load at the sex locus. Nat. Ecol. Evol. 1, 0011 (10.1038/s41559-016-0011) 28812560

[31] Zayed A, Packer L 2001 High levels of diploid male production in a primitively eusocial bee (Hymenoptera: Halictidae). Heredity 87, 631–636. (10.1046/j.1365-2540.2001.00952.x) 11903558

[32] Takahashi J, Ayabe T, Mitsuhata M, Shimizu I, Ono M 2008 Diploid male production in a rare and locally distributed bumblebee, *Bombus florilegus*. Insectes Soc. 55, 43–50. (10.1007/s00040-007-0976-z)

[33] Boff S, Soro A, Paxton RJ, Alves-dos-Santos I 2014 Island isolation reduces genetic diversity and connectivity but does not significantly elevate diploid male production in a neotropical orchid bee. Conserv. Genet. 15, 1123–1135. (10.1007/s10592-014-0605-0)

[34] Soro A, Quezada-Euan JJG, Theodorou P, Moritz RFA, Paxton RJ 2017 The population genetics of two orchid bees suggests high dispersal, low diploid male production and only an effect of island isolation in lowering genetic diversity. Conserv. Genet. 18, 607–619. (10.1007/s10592-016-0912-8)

[35] van Nouhuys S, Hanski I 2005 Metacommunities of butterflies, their host plants and their parasitoids. In Metacommunities: spatial dynamics and ecological communities, (eds Holyoak M, Leibold MA, Holt RD), pp. 99–121. Chicago, IL: University of Chicago Press.

[36] Ojanen SP, Nieminen M, Meyke E, Poyry J, Hanski I 2013 Long-term metapopulation study of the Glanville fritillary butterfly (*Melitaea cinxia*): survey methods, data management, and long-term population trends. Ecol. Evol. 3, 3713–3737. (10.1002/ece3.733) 24198935 PMC3810870

[37] Hanski I, Schulz T, Wong SC, Ahola V, Ruokolainen A, Ojanen SP 2017 Ecological and genetic basis of metapopulation persistence of the Glanville fritillary butterfly in fragmented landscapes. Nat. Commun. 8, 14504 (10.1038/ncomms14504) 28211463 PMC5321745

[38] Kahilainen A, van Nouhuys S, Schulz T, Saastamoinen M 2018 Metapopulation dynamics in a changing climate: increasing spatial synchrony in weather conditions drives metapopulation synchrony of a butterfly inhabiting a fragmented landscape. Glob. Change Biol. 24, 4316–4329. (10.1111/gcb.14280) PMC6120548 29682866

[39] Nair A, Fountain T, Ilkonen S, Ojanen SP, van Nouhuys S 2016 Spatial and temporal genetic structure at the fourth trophic level in a fragmented landscape. Proc. R. Soc. B. 283, 20160668 (10.1098/rspb.2016.0668) PMC4892806 27226470

[40] Montovan KJ, Couchoux C, Jones LE, Reeve HK, van Nouhuys S 2015 The puzzle of partial resource use by a parasitoid wasp. Am. Nat. 185, 538–550. (10.1086/680036) 25811087

[41] Couchoux C, Seppä P, van Nouhuys S 2016 Strong dispersal in a parasitoid wasp overwhelms habitat fragmentation and host population dynamics. Mol. Ecol. 25, 3344–3355. (10.1111/mec.13696) 27159020

[42] van Nouhuys S, Ehrnsten J 2004 Wasp behavior leads to uniform parasitism of a host available only a few hours per year. Behav. Ecol. 15, 661–665. (10.1093/beheco/arh059)

[43] Nair A, van Nouhuys S 2015 Microsatellite markers for a hyperparasitoid wasp from a fragmented landscape. Conserv. Genet. Resour. 7*,* 565–568. (10.1007/s12686-015-0425-7)

[44] Rousset F 2008 genepop'007: a complete re-implementation of the genepop software for Windows and Linux. Mol. Ecol. Resour. 8, 103–106. (10.1111/j.1471-8286.2007.01931.x) 21585727

[45] Nair A, Fountain T, Ikonen S, Ojanen SP, van Nouhuys S 2016 Data from: Spatial and temporal genetic structure at the fourth trophic level in a fragmented landscape. *Dryad Digital Repository*. (10.5061/dryad.51j99) PMC4892806 27226470

[46] Nair A, Nonaka E, van Nouhuys S 2018 Data from: Increased fluctuation in a butterfly metapopulation leads to diploid males and decline of a hyperparasitoid *Dryad Digital Repository*. (10.5061/dryad.56qf11h) PMC6125898 30135149

[47] Travis JMJ, Murrell DJ, Dytham C 1999 The evolution of density-dependent dispersal. Proc. R. Soc. Lond. B 266, 1837–1842. (10.1098/rspb.1999.0854)

[48] Poethke HJ, Hovestadt T 2002 Evolution of density- and patch-size-dependent dispersal rates. Proc. R. Soc. Lond. B 269, 637–645. (10.1098/rspb.2001.1936) PMC1690934 11916481

[49] Hasselmann M, Gempe T, Schiøtt M, Nunes-Silva CG, Otte M, Beye M 2008 Evidence for the evolutionary nascence of a novel sex determination pathway in honeybees. Nature 454, 519–522. (10.1038/nature07052) 18594516

[50] Cornuet JM 1980 Rapid estimation of the number of sex alleles in panmictic honeybee populations. J. Apic. Res. 19, 3–5. (10.1080/00218839.1980.11099991)

[51] Ruf D, Dorn S, Mazzi D 2011 Females leave home for sex: natal dispersal in a parasitoid with complementary sex determination. Anim. Behav. 81, 1083–1089.

[52] Thiel A, Weeda AC, de Boer JG, Hoffmeister TS 2013 Genetic incompatibility drives mate choice in a parasitic wasp. Front. Zool. 10, 43 (10.1016/j.anbehav.2011.02.028) 23895372 PMC3734144

[53] Metzger M, Bernstein C, Hoffmeister TS, Desouhant E 2010 Does kin recognition and sib-mating avoidance limit the risk of genetic incompatibility in a parasitic wasp? PLoS ONE 5, e13505 (10.1371/journal.pone.0013505) 20976063 PMC2957437

[54] Beukeboom LW, van de Zande L 2010 Genetics of sex determination in the haplodiploid wasp *Nasonia vitripennis* (Hymenoptera: Chalcidoidea). J. Genet. 89, 333–339. (10.1007/s12041-010-0045-7) 20877000

[55] Niyibigira EI, Overholt WA, Stouthamer R 2004 *Cotesia flavipes* Cameron and *Cotesia sesamiae* (Cameron) (Hymenoptera: Braconidae) do not exhibit complementary sex determination: evidence from field populations. Appl. Entomol. Zool. 39, 705–715. (10.1303/aez.2004.705)

[56] Geuverink E, Verhulst EC, van Leussen M, van de Zande L, Beukeboom LW 2018 Maternal provision of non-sex-specific *transformer* messenger RNA in sex determination of the wasp *Asobara tabida*. Insect Mol. Biol. 27, 99–109. (10.1111/imb.12352) 29030993

[57] Frankham R 1997 Do island populations have less genetic variation than mainland populations? Heredity 78, 311–317. (10.1038/hdy.1997.46) 9119706

[58] Ross KG, Vargo EL, Keller L, Trager JC 1993 Effect of founder event on variation in the genetic sex-determining system of the fire ant *Solenopsis invicta*. Genetics 135, 843–854. 8293983 10.1093/genetics/135.3.843 PMC1205724

[59] Tsuchida K, Kudô K, Ishiguro N 2014 Genetic structure of an introduced paper wasp, *Polistes chinensis antennalis* (Hymenoptera, Vespidae) in New Zealand. Mol. Ecol. 23, 4018–4034. (10.1111/mec.12852) 25041373

[60] Lande R 1993 Risks of population extinction from demographic and environmental stochasticity and random catastrophes. Am. Nat. 142, 911–927. (10.1086/285580) 29519140

[61] Saccheri I, Kuussaari M, Kankare M, Vikman P, Fortelius W, Hanski I 1998 Inbreeding and extinction in a butterfly metapopulation. Nature 392, 491–494. (10.1038/33136)

[62] Thrall PH, Richards CM, McCauley DE, Antonovics J 1998 Metapopulation collapse: the consequences of limited gene-flow in spatially structured populations. In Modeling spatiotemporal dynamics in ecology (eds Bascompte J, Soulé RV), pp. 79–100. Berlin, Germany: Springer.

[63] Hufbauer RA, Szucs M, Kasyon E, Youngberg C, Koontz MJ, Richards C, Tuff T, Melbourne BA 2015 Three types of rescue can avert extinction in a changing environment. Proc. Natl Acad. Sci. USA 112, 10 557–10 562. (10.1073/pnas.1504732112) 26240320 PMC4547288

[64] Fujii S, Kubo K, Takayama S 2016 Non-self- and self-recognition models in plant self-incompatibility. Nat. Plants 2, 16130 (10.1038/nplants.2016.130) 27595657

[65] Castric V, Vekemans X 2004 Plant self-incompatibility in natural populations: a critical assessment of recent theoretical and empirical advances. Mol. Ecol. 13, 2873–2889. (10.1111/j.1365-294X.2004.02267.x) 15367105

[66] Hanski I, Gaggiotti O 2004 Ecology, genetics, and evolution of metapopulations. San Diego, CA: Elsevier Academic Press.

[67] Zayed A, Roubik DW, Packer L 2003 Use of diploid male frequency as an indicator of pollinator decline. Biol. Lett. 271, 9–12. (10.1098/rsbl.2003.0109) PMC1809989 15101404

