# Supplementary material for: Increased fluctuation in a butterfly metapopulation leads to diploid males and decline of a hyperparasitoid
Source: Proc Biol Sci. 2018 Aug 22;285(1885):20180372. doi: 10.1098/rspb.2018.0372 (PMC6125898; doi:10.1098/rspb.2018.0372)
Supplement: Model description [file rspb20180372supp1.pdf]

Electronic Supplementary material

Increased fluctuation in a butterfly metapopulation leads to diploid males and decline of hyperparasitoid

Abhilash Nair, Etsuko Nonaka, Saskya van Nouhuys

Model description

Model overview — We developed a simulation model of the population and genetic dynamics of the hyperparasitoid in response to the increasing amplitude of fluctuation of local butterfly population sizes and increasing spatial correlation among them. The model is a discrete-time, spatially-implicit metapopulation model with three trophic levels (the butterfly, the parasitoid, and the hyperparasitoid), parameterized using the known long-term butterfly dynamics [36,38] and parasitoid population dynamics, behaviour, genetic structure and natural history [35, 39, 40]. The definitions of state variables and parameters, and parameter values and their sources are listed in Table S1. The model was implemented in MATLAB R2017a, and the code is available from the Dryad Digital Repository: https://doi.org/10.5061/dryad.56qf11h

The spatial structure — We divided Åland into 12 sub-regions to represent realistic heterogeneity in host availability to the hyperparasitoid arising from spatial distribution of habitat patches for the butterfly. The sub-regions were based on the 12 butterfly survey areas in Åland [36], which are delineated by topography (figure S1). In the model, the hyperparasitoid population is spatially structured with dispersal, while dispersal of the butterfly and the parasitoid is not modeled. Although the hyperparasitoid is known to be dispersive based on the spatial genetic structure of the population [39], individual wasps are dispersal limited

due to small body size (~ 6 mm) and the heterogeneity of host availability in the fragmented landscape. We estimated the butterfly larval abundance in these sub-regions using the autumn survey [36] data from 2003 to 2016. The populations in 4 sub-regions were small (average number of larvae < 3000), 4 were medium (between 3000 and 6000), and 4 were large (> 10000). These sub-regions were arranged in a 4x3 lattice with links to 4 neighboring regions. An individual hyperparasitoid can disperse once before mating, at a constant dispersal probability, h. We used reflecting boundaries along the lattice boundaries, as wasps are unlikely to fly toward water or forest. We intend the model to be a general, spatially implicit model of a spatially structured population in a heterogeneous landscape, so the locations of the sub-regions were randomized in each simulation to avoid peculiarities arising from a particular configuration. Because female parasitoids tend to oviposit eggs within 2 km [39], we used 2-dimensional stepping-stone dispersal [46,47] to capture the dispersive nature of the hyperparasitoid. Hence, emigrating individuals go to any of the sub-regions directly connected to the natal sub-region with equal probability.

The butterfly larvae sub-model (Melitaea cinxia) — The mean population size of butterfly larvae at time t in sub-region i, $\bar{B}_{i,t}$, is drawn from a multivariate log-normal distribution with the means and the variance-covariance matrix of the associated multivariate normal distribution estimated from the autumn survey data collected between 2003 and 2009 [36]. We consider this period to represent a standard level of population fluctuations and later use this as the baseline to simulate increased variability. The calculation takes into account a 60% detection rate of the larval nests by surveyors [36] and the average number of larvae emerging from each nest in spring (L = 18 per nest), based on annual counts of the larvae each spring after winter diapause. The actual number of larvae in sub-region i, $B_{i,t}$, was determined by drawing values from a Poisson distribution with mean equal to $\bar{B}_{i,t}$.

We simulated variability in population fluctuations by varying the cross-correlation coefficient, ρ, and the standard deviations in the calculation of the variance-covariance matrix for the population dynamics of the 12 sub-regions. The value of ρ was constant across the sub-regions, while the standard deviations were estimated for each region from the fall survey data collected between 2003 and 2009. The correlation in the nest counts among the sub-regions increased from about 0.25 to about 0.75 and their standard deviations by 1.85-fold between 2003-2009 and 2010-2016 (see figure. 3). In the simulations, we varied ρ and the factor multiplying the standard deviations, M, to simulate variability in the butterfly larvae populations (Table S1). In the parameter space, $\rho = 0.25$ and $M = 1$ correspond to the condition in 2003-2009, and $\rho = 0.75$ and $M = 1.85$ to the situation in 2010-2016 (see figure 3).

The host parasitoid sub-model (Hyposoter horticola) — The population size of the parasitoid before hyperparasitism at time t in sub-region i, $N'_{i,t}$, is drawn from a binomial distribution with the butterfly larval population size as the sample size and probability of success equal to $\frac{1}{3}$ [40,42]. We assumed no additional mortality of the parasitoid.

We modeled the mean rate of hyperparasitism at time t in sub-region i, $\bar{f}_{i,t}$, as a function of the ratio between hyperparasitoid female and parasitoid densities, and visually fit a saturating function to field data (figure S2),

$$\bar{f}_{i,t} = a\left(1 - e^{-b\frac{F_{i,t}}{N'_{i,t}}}\right) \qquad \text{(E1)}$$

where $N'_{i,t}$ denotes the parasitoid population size before hyperparasitism and $F_{i,t}$ is the female hyperparasitoid population size at time t in sub-region i. The parameter a corresponds to the maximum rate of parasitism and b corresponds to the rate of increase in the rate of parasitism as the ratio between the densities of hyperparasitoid females and parasitoids increases. Therefore, the rate of hyperparasitism varied as the hyperparasitoid-parasitoid ratio changed

over time. We did not statistically fit the function to the data, as the data are limited and the fitted function looked unreasonably low. Because other mechanisms affecting the population of the hyperparasitoid are scarcely known, we preferred a simple model to focus on main processes involved in hyperparasitism. We thus used the empirical data as a guide to estimate the parameters because the functional form is theoretically reasonable and the data set is not large. While fitting the simulation outcomes to the data, we ascertained that the simulated rates of hyperparasitism covered the variation in the field data, that the simulated proportion of diploid male hyperparasitoids matched the value observed in the sample in 2008 (9%), and that the simulated population sizes of the two parasitoids were similar to the empirical data. We consider the parameterization of the function to be conservative with respect to extinction risk of the hyperparasitoid. It allows greater population size of the hyperparasitoid than it would have been if we had used parameter values from a statistically fitted function (not shown), as there is no feedback to the parasitoid population (i.e., the number of parasitoids available for hyperparasitism is unaffected by the population size of the hyperparasitoid in the previous year). To reflect stochastic variation in this relationship from year to year, the actual rate of hyperparasitism was drawn from a normal distribution with the mean equal to $\bar{f}_{i,t}$ with standard deviation 0.1 to match the scatter seen in the empirical data (figure S2)

$$f_{i,t} = Normal(\bar{f}_{i,t}, 0.1). \qquad \text{(E2)}$$

The number of actually parasitized hosts at time t, $N_{i,t}^{p}$ is drawn from a binomial distribution with probability of success equal to $f_{i,t}$ and the number of trials equal to the number of parasitoids. Hence, the remaining healthy parasitoids is $N_{i,t} = N'_{i,t} - N_{i,t}^{p}$.

The hyperparasitoid sub-model (M. cf. *stigmaticus)* — To simulate the hyperparasitoid population dynamics influenced by the CSD alleles, we developed an individual-based simulation model in which each individual is represented by one sex locus. Normal males are

haploid, and females are diploid. Homozygous diploids are removed from the female population and categorized as diploid males. We assume diploid males to be sterile, as species with fertile diploid males are uncommon [12,13]. This sub-model keeps track of the abundances of female, male, and diploid male hyperparasitoids.

This sub-model is composed of several probabilistic rules governing mortality, dispersal, mating, and reproduction of hyperparsitoid individuals. The hyperparasitoid population suffers from a constant, indiscriminate mortality every generation before mating (*Mesmort*, Table S1). Individuals may disperse before mating from each region to one of the neighboring sub-regions with probability h. Nair *et al*. [39] indicates possible variation in migration rate among years, and therefore we used three levels of migration rate (0.002, 0.014 (estimated mean), 0.027) to account for the uncertainties (Table S1). We calibrated the value of *h* by counting the number of successful migrants (migrated and successfully reproduced) in each generation.

After dispersal, the hyperparasitoid mates in the destination sub-region or in the natal sub-region if it does not disperse. Each female randomly chooses one male in the same sub-region, while males can mate with multiple females if they are chosen multiple times. We assumed no difference in the probability of mating between normal and diploid males as they appear morphologically identical. The total number of offspring at time *t* in sub-region *i* is $N_{i,t}^{p}$, the number of hyperparasitized parasitoids (i.e., one hyperparasitoid emerges from one parasitoid in *Mesochorus*). Parents are randomly assigned to each offspring from the list of mated pairs with replacement. Because females mated with diploid males can have only sons (eggs are not fertilised), their probability of having offspring is reduced by $(1 - s_0)$, where s_0 is the mothers' intended sex ratio (the fraction of sons). The sex of offspring is female if the mother intends to produce a daughter, her mate is a normal male, and offspring is heterozygous at the sex locus. If homozygous, the offspring are diploid males. It is male if the mother intends

to produce a son. Male offspring receive one allele randomly selected from the mother, while daughters inherit one allele randomly selected from the mother and receive the other one from the father. Each allele has a probability of 10^{-7} to mutate into a new allele [25,48]. At this point, diploid males are identified and removed from the pool of diploid offspring.

Simulation experiments — We ran 30 replicates for each parameter set, and each replicate was run for 10,000 generations to ensure that transient dynamics disappeared. We initialized the genetics of the populations with 10 CSD alleles, as founder populations have been shown to have comparable number of CSD alleles [30] and also the method presented in Cornuet [49] estimates 10 alleles for N_e of 500 and mutation rate of 10^{-7}. The number of unique alleles at the four microsatellite loci in *Mesochorus* ranged from 6 to 8 [39]. In the simulations, 10 CSD alleles maintained 9-10% of diploid males in the population under the level of fluctuation in butterfly population size that occurred during the time period of 2003-2009 (h = 0.014, intermediate). This matches the empirically determined proportion of diploid males in 2008-2009. We initialized the model with 900 female hyperparasitoids, a rate of hyperparasitism equal to 0.6, and the number of parasitoids equal to $\frac{1}{3}$ of the number of butterflies in the initial year. We varied the multiplier on the level of fluctuation, *M*, to cover a parameter region from zero to three times the baseline (2009) fluctuation of the butterfly population sizes. The correlation parameter ρ was varied from 0 to 1. The correlation of zero means the local population dynamics of the butterfly are asynchronised, while that of one means they are synchronised. We summarized the data from the latter half of simulations for the hyperparasitoid at the whole Åland scale by recording the number of simulations in which the entire population went extinct, and for each replicate the minimum number of CSD alleles, mean proportion of diploid males among all the males, the mean number of occupied sub-regions, and mean total population size. At the sub-regional scale, for each replicate we calculated extinction rate (the number of local extinction events divided by the number of years

present), the minimum number of CSD alleles, and the fraction of diploid males averaged over the latter half of simulation period. To elucidate the cost of diploid male production separate from demographic effects, we compared the model outcomes under sl-CSD conditions described above with a hypothetical situation in which sl-CSD was absent so that all diploid offspring develop into functional females, and therefore there is no cost of diploid male production.

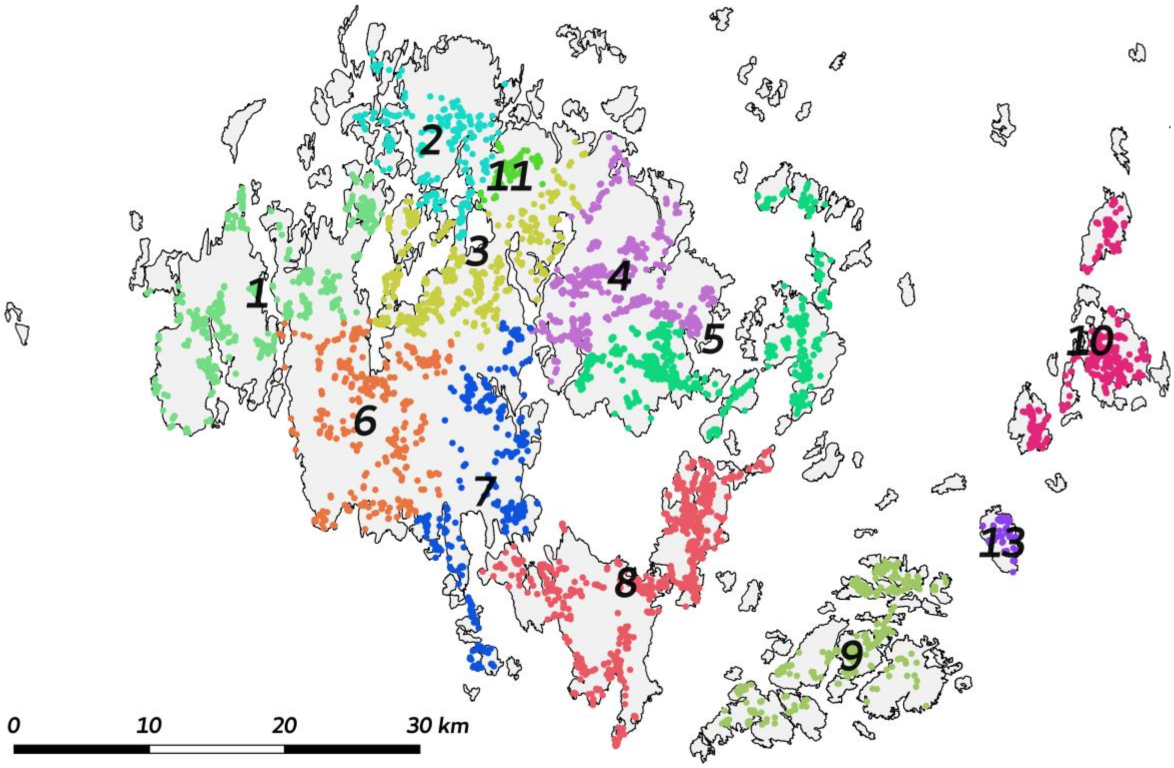

Figure S1. A map of the main Åland islands. The dots represent butterfly habitat patches, and survey areas are coded by color. The numbers are the survey area IDs used in the autumn survey (Survey areas 12, 14, and 15 were excluded as no butterfly nests were recorded for the study period of this study (2003-2016)).

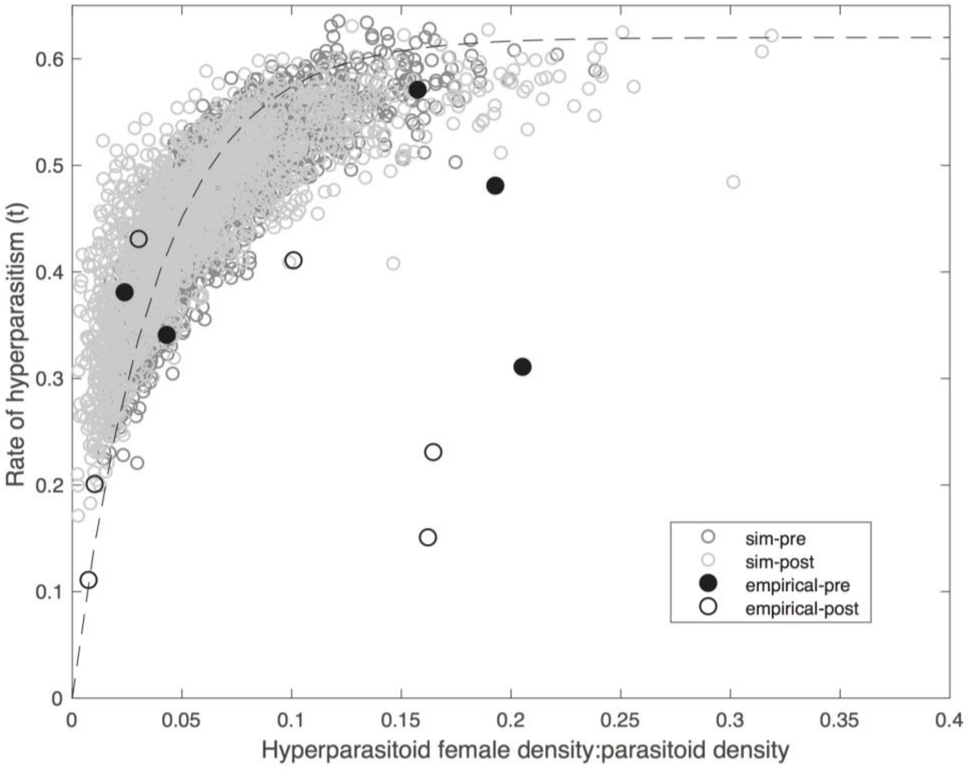

Figure S2. The observed rate of hyperparasitism (open black circles for post regime shift and filled black circles for pre regime shift) plotted against the ratio of the population sizes of female hyperparasitoids (*F*) and parasitoids (*N*). The grey circles are from simulations (the dark grey is pre regime change and light grey is post regime change). The data are from years between 2003 and 2016. The dashed line represents the function used to model the rate of hyperparasitism $\left(f(t) = 0.62\left(1 - e^{-26\frac{F}{N}}\right)\right)$. E3

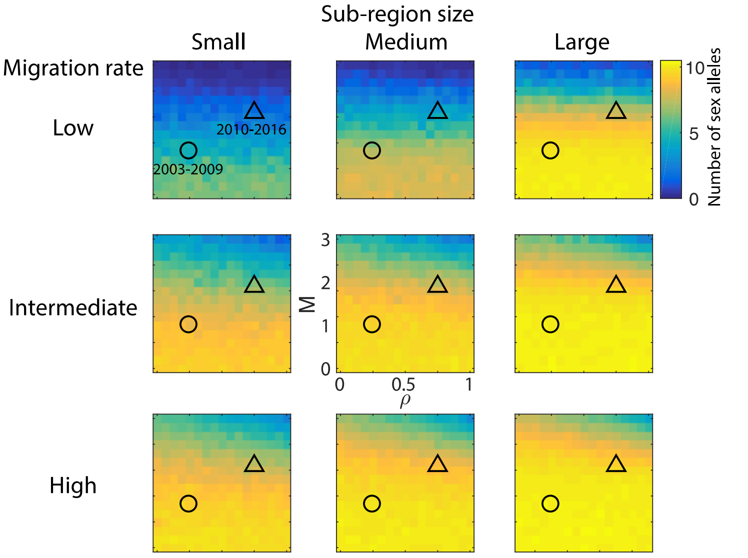

S3A

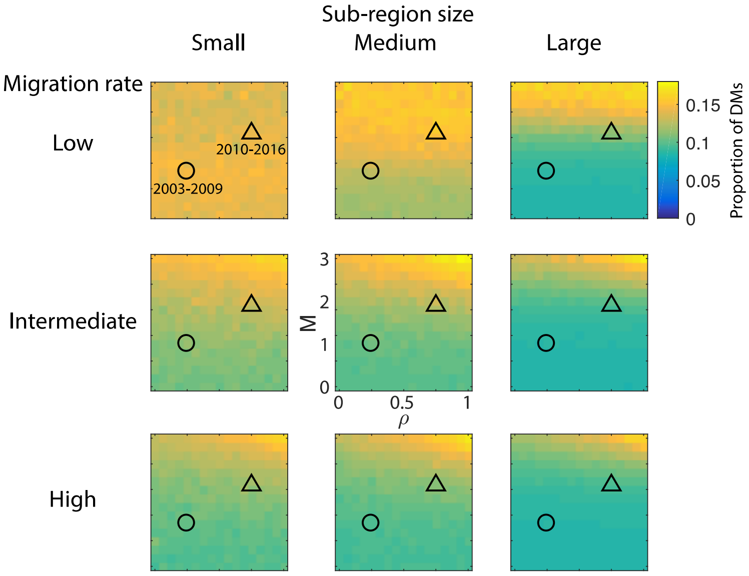

S3B

Figure S3. The number of hyperparasitoid *sl*-CSD alleles (A) and the proportion of diploid males (B) with varying cross-correlation among local butterfly populations (ρ) and fluctuation multiplier (M), at three levels of hyperparasitoid migration rate in small, medium, and large sub-region.

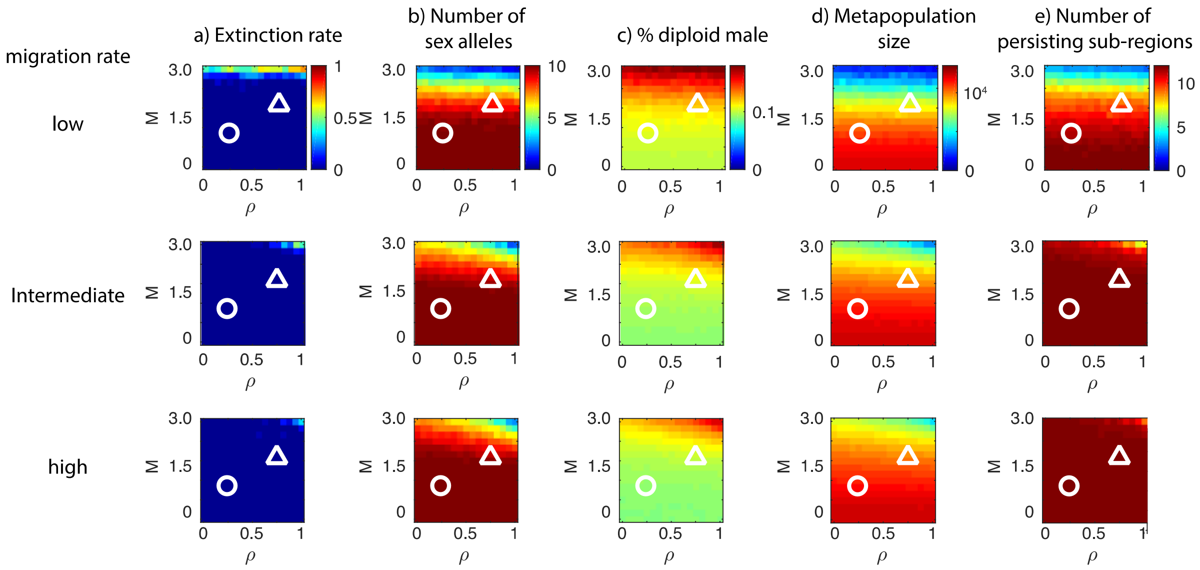

S4A.

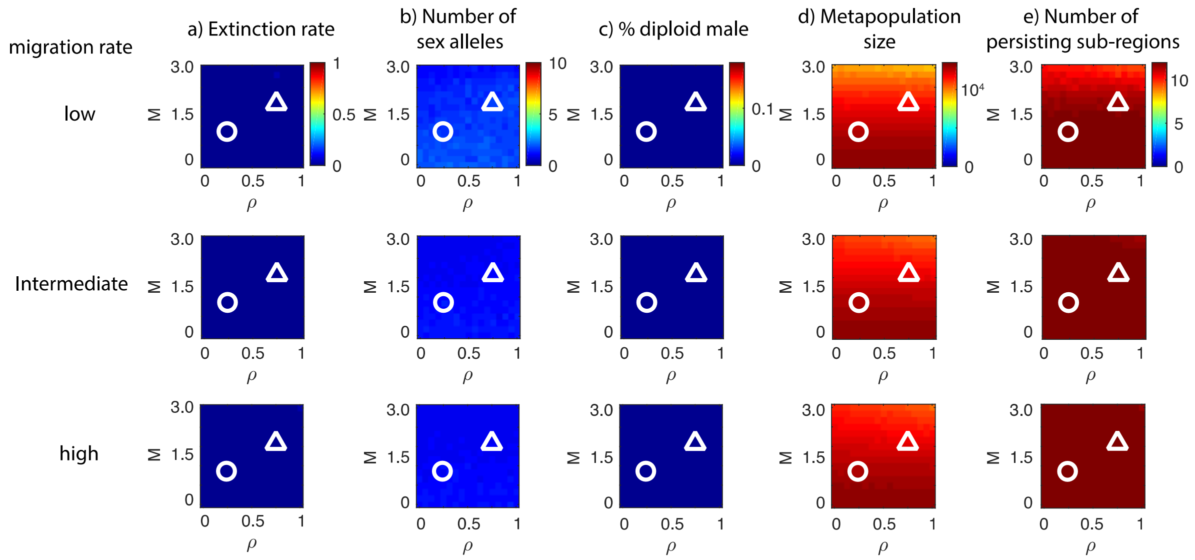

S4B.

Figure S4. Model outputs at the Åland scale for the scenario with CSD on (panel A) and off (panel B) at three *hyperparasitoid* migration rates. From left to right, extinction rate (a), number of sex alleles (b), proportion of diploid males (c), population size (total number of individuals; d), and the number of persisting local populations (occupied sub-regions; e). For the scenario with CSD off (panel B), there are no diploid males by definition, so the value in c) is uniformly 0, but it is presented in the figure for consistency between the two sets of panels.

Table S1. The names and definitions of the state variables and the parameters in the model. The subscripts *i* and *t* signify sub-regions and time step, respectively.

State variables:

Name	Description
$B_{i,t}(\bar{B}_{i,t})$	Larval population size (mean)
$N_{i,t}(N'_{i,t})$	Parasitoid population size after (before) hyperparasitism
$\bar{N}_{i,t}$	Mean parasitoid population size before hyperparasitism
$N^{p}_{i,t}$	The number of parasitized parasitoids
$f_{i,t}(\bar{f}_{i,t})$	(Mean) rate of hyperparasitism
$F_{i,t}$	The number of female hyperparasitoids
$M_{i,t}$	The number of male hyperparasitoids
$DM_{i,t}$	The number of diploid male hyperparasitoids

Model parameters:

Name	Description	Value	Explanations
CSD_0	Initial number of CSD alleles	10	Adjusted to produce 9-10% diploid males under the fluctuation regime between 2003 and 2009. Also see [49]
μ	Mutation rate at the CSD locus	10^{-7}	[25,48]
s_0	Fraction of male offspring intended by mothers	0.5	Assuming 50:50 sex ratio intended by mothers
h	Migration rate	0.002, 0.014, 0.027	To match the estimate for N_m from GENEPOP [44]. The lower and higher values are also included to reflect uncertainty in the estimate. These roughly correspond to 5, 36, and 72 successful migrants on average for the entire study area.
σ_p	Standard deviation of the normal distribution for stochastic variation in the rate of parasitism	0.1	Function for rate of parasitism
a	The asymptotic value of the function for the rate of parasitism	0.62	Function for rate of parasitism
b	Parameter controlling the rising slope of the asymptotic function	26	Function for rate of parasitism

Mesomort	Hyperparasitoid mortality	2/3	Individuals are randomly removed
Hypomort	Parasitoid mortality other than parasitism	0	
L	The average number of larvae per nest in spring	18	van Nouhuys, *unpublished data*
M	Multiplier on the standard deviation of larval population size estimated from the fall survey data	Varied from 0 to 3 by an increment of 0.2	
ρ	Mean cross-correlation between larval population size among the 12 survey areas	Varied from 0 to 1 by an increment of 0.0625	
p	Probability that diploid males mate, relative to normal male	1	It is 1 because normal and diploid males appear morphologically similar.
F_0	Initial number of female hyperparasitoids	900	
f_0	Initial rate of hyperparasitism	0.6	
g	Rate of parasitism of larvae	1/3	
